# Supplementary material for: A Global Screen for Assembly State Changes of the Mitotic Proteome by SEC-SWATH-MS
Source: Cell Syst. 2020 Feb 26;10(2):133–155.e6. doi: 10.1016/j.cels.2020.01.001 (PMC7042714; doi:10.1016/j.cels.2020.01.001)

O15511 | ARPC5_HUMAN | ARPC5 ARC16
Monomer MW [kDa]: 16.32 Monomer expected elution fraction: 56

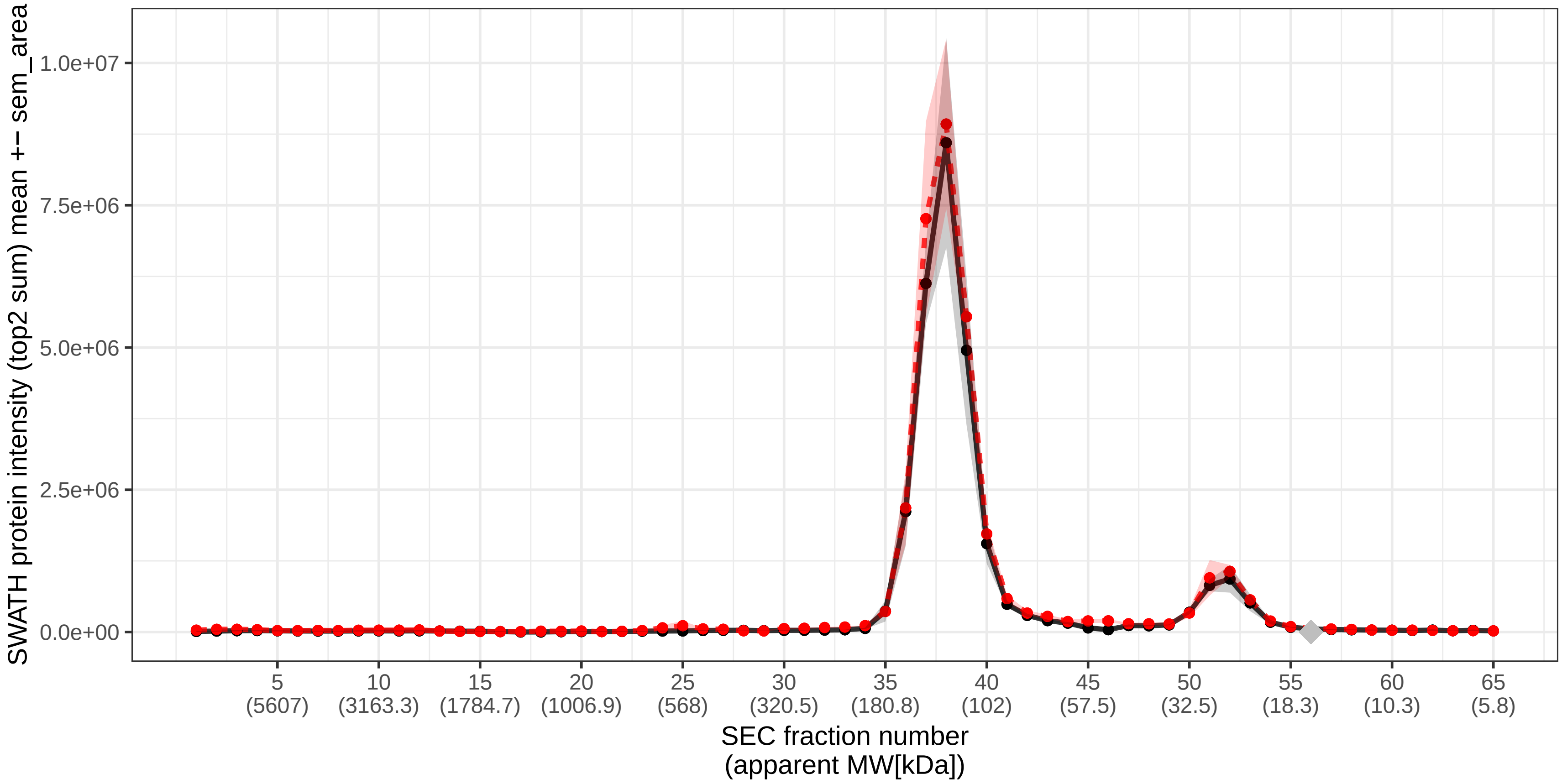

Supplement: Data S1. SEC-SWATH-MS Protein Chromatograms, Related to Figure 1 [file mmc6.zip › SECchrom_O15511_ARPC5_HUMAN_ARPC5_ARC16.pdf]
